# Supplementary material for: Predicting visual function by interpreting a neuronal wiring diagram
Source: Nature. 2024 Oct 2;634(8032):113–23. doi: 10.1038/s41586-024-07953-5 (PMC11446822; doi:10.1038/s41586-024-07953-5)
Supplement: Supplementary file 6 — CRF and ERF predictions for individual TmY4 and TmY9 cells. Analogous to Supplementary Data 3, but for TmY target types. Shown are the top four monosynaptic pathways, the strongest pathway passing through each of the top ten intermediary types (ranking from Extended Data Fig. 7), and the trisynaptic pathway Tm1–TmY–Dm3–TmY (see the section entitled Prediction of spatial normalization). [file 41586_2024_7953_MOESM6_ESM.zip › DataS4/TmY4/720575940617193147.pdf]

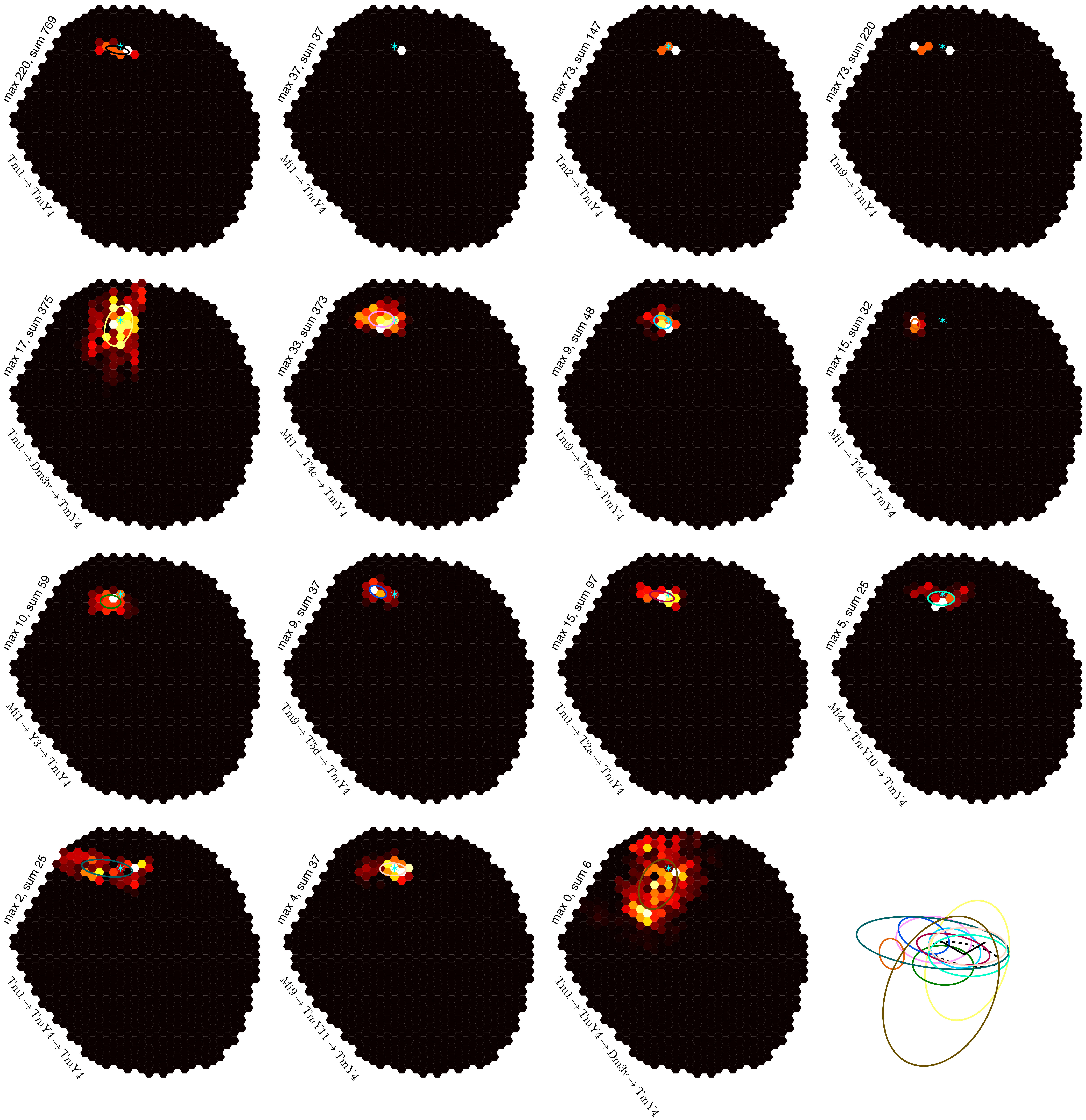

max 220, sum 769
Tm1 → TmY4
max 37, sum 37
Mi1 → TmY4
max 73, sum 147
Tm2 → TmY4
max 73, sum 220
Tm9 → TmY4
max 17, sum 375
Tm1 → Dm3v → TmY4
max 33, sum 373
Mi1 → T4c → TmY4
max 9, sum 48
Tm9 → T5c → TmY4
max 15, sum 32
Mi1 → T4d → TmY4
max 10, sum 59
Mi1 → Y3 → TmY4
max 9, sum 37
Tm9 → T5d → TmY4
max 15, sum 97
Tm1 → T2a → TmY4
max 5, sum 25
Mi4 → TmY10 → TmY4
max 2, sum 25
Tm1 → TmY4 → TmY4
max 4, sum 37
Mi9 → TmY11 → TmY4
max 0, sum 6
Tm1 → TmY4 → Dm3v → TmY4
